# Local hydrodynamics at edges of marine canopies under oscillatory flows

**DOI:** 10.1371/journal.pone.0201737

**Published:** 2018-08-22

**Authors:** Teresa Serra, Carolyn Oldham, Jordi Colomer

**Affiliations:** 1 Department of Physics, University of Girona, Girona, Spain; 2 Department of Civil, Environmental and Mining Engineering, The University of Western Australia, Perth WA, Australia; Coastal Carolina University, UNITED STATES

## Abstract

Canopy fragmentation increases both spatial heterogeneity and patch edges which, in turn, is then likely to modify the local hydrodynamics in the canopy. The orientation of the edge versus the wave and current field is also expected to play an important role in determining wave attenuation and sheltering at the edge of a canopy. We investigated the effect a longitudinal edge (i.e. with its main axis aligned to wave direction) of a simulated canopy has on local edge hydrodynamics. The effect that both canopy density and flexibility have on the hydrodynamics was studied. Flexible plants reduced the wave velocity and the turbulent kinetic energy with distance into the canopy and this attenuation increased as the density of the canopy increased. Compared to flexible plants, an edge of rigid plants produced a higher wave velocity attenuation coupled with an increase in the turbulent kinetic energy with distance into the canopy despite having the same canopy density. This greater wave attenuation at the edge coincided with the shifting of the associated mean current that, in turn, produced an increase in the turbulent kinetic energy at the edge in the canopy. The effect was accentuated when the canopy density increased. The wave velocity attenuation was a linear function of the canopy cover. While flexible plants reduced the turbulent kinetic energy following a linear function of the canopy cover, rigid canopies increased the turbulent kinetic energy following a linear function of the canopy cover. In the case of the flexible vegetation, the lengths of both the inner and outer canopy boundary layers increased as the canopy cover increased.

## Introduction

Aquatic vegetation plays a critical role in protecting coastal areas. Coastal meadows of aquatic vegetation support infauna [1,2], store carbon [3], reduce erosion [4] and stabilize sediment beds [5], but there are still gaps in the knowledge about the conditions that optimize this function [6]. Bed stabilization in submerged coastal canopies is related to the absorption of kinetic energy by vegetation through a reduction in turbulence [7–10], waves [8,11–13], and mean currents [14,15], and depends on plant flexibility [16], the submergence ratio and canopy density [10,17,18]. As such, unidirectional flow through vegetated canopies has been described by several authors [19,20], where a reduction of up to 90% of the mean flow velocity has been observed. However, many seagrass meadows are situated in regions dominated by oscillatory flows rather by unidirectional currents. Several authors [4,8,12,13] have investigated the attenuation of both wave velocity and turbulent kinetic energy within a laboratory simulated canopy. Pujol et al [8] compared the ability of flexible and rigid plants to reduce vertical wave velocity and turbulent kinetic energy. While flexible plants always reduced the turbulent kinetic energy, rigid plants with high canopy densities produced an increase in the turbulent kinetic energy in the top layer of the canopy. Wave velocity attenuation by seagrass meadows has been also studied [11,21–23]. A maximum reduction in the wave velocity between 20% and 30% was found inside coastal meadows, compared to immediately above the meadow [22,23]. As a consequence of wave attenuation by canopies, sediment resuspension is reduced [4,5,14,24,25] and particles became trapped, thus increasing water transparency which has an impact on the available light within the canopy [26,27] as well as its global health.

As a result of anthropogenic activities such as anchoring, trawling, fish farming and cables and pipes being laid [28] and also due to the effects of climate change effects [29], total seagrass cover has declined worldwide. As a result canopy fragmentation occurs because mosaics of seagrass patches or canopies with gaps are generated [30]. Low shoot densities, high mortality rates and high fragmentation (the patchiness of continuous habitats) are all indicators of seagrass meadow degradation [31]. Gaps within a canopy leave the bed exposed to both waves and currents and, as such, as they are no longer protected from erosion [32,33] they produce more turbid waters with lower amounts of available light than those in regions covered with vegetation [27,34,35]. The orientation of the canopy gaps (i.e. relative to wave and current direction) is expected to impact the extent of such erosion. The little work that has been done examining differences in orientation suggest that both gap widths and canopy density control the extent of canopy sheltering and therefore the impact the gap’s orientation has on erosion. The presence of longitudinal (parallel to the wave/current direction) gaps in a canopy of flexible plants produce a wave penetration within the lateral vegetation, with the extent of penetration being dependent on canopy gap width and canopy density [36]. The presence of transversal (perpendicular to the wave/current direction) gaps within a canopy, produces both wave and turbulent kinetic energy penetration into the nearby lateral vegetation [37]. In both cases, the degree of wave penetration and turbulent kinetic energy attenuation depends on the density of the canopy and the width of the gap. Furthermore, lateral vegetation protects the gap although to what extent depends on both the density of the canopy and the width of the gap [37]. Folkard [38] studied the hydrodynamics in canopies of flexible plants with the presence of transversal gaps and under a unidirectional flow. In his study, the gap aspect ratio and the Reynolds number are the main parameters in determining the type of flow through the canopy and also in which cases the canopy protects the gap.

This work highlighted that canopy fragmentation can lead to habitats that are more vulnerable to external pressures than continuous canopies are. Fragmentation might leave small patches of vegetation disconnected from the canopies. Small patches will have lower canopy densities, shorter leaves and lower nutrient stores than continuous canopies [39], possibly due to the impact of stronger waves and currents experienced within the small patches compared to the large ones.

Fragmentation results in an increase in the number and/or extent of canopy edges and a decrease in inner canopy regions [40]; these edges will experience hydrodynamics that are different to that found within the inner canopy regions. Both waves and turbulence are gradually attenuated across the edge of a seagrass coastal meadow [21]. Nutrient uptake is greatest near the edge of seagrass patches [41], where currents and turbulence are higher than within the canopy. Bowden et al [42] proposed edges as transitional regions between bare sediments and the meadow itself. Ricart et al [43] found that the carbon stocks are 20% higher inside seagrass patches than at seagrass edges. The presence of an edge of rigid vegetation under unidirectional flow has been studied by several authors [44,45], who found that the mean flow is gradually reduced across the edge of the vegetation (i.e. from the bare soil towards the inner part of the vegetation), producing a shear layer at the canopy edge. Little work has been undertaken investigating hydrodynamics along edges under oscillatory flows.

This study aims to determine the hydrodynamics at canopy edges under an oscillating flow, focusing on canopy edges orientated longitudinally (parallel) to the direction of wave propagation. Furthermore, our study aims to evaluate the horizontal length of the characteristic regions that define the hydrodynamics across the boundary of a canopy of flexible plants. To determine the effect plant stiffness has at the edge of a canopy, the hydrodynamics at the edge of a flexible canopy are compared with those at the edge of a rigid canopy. The results obtained from laboratory experiments are also compared to those obtained in coastal canopies of *Posidonia oceanica* meadows. Here, canopy cover (instead of the canopy density) is considered to compare the laboratory results of our study with those of the coastal canopies.

## Materials and methods

**Fig 1:**
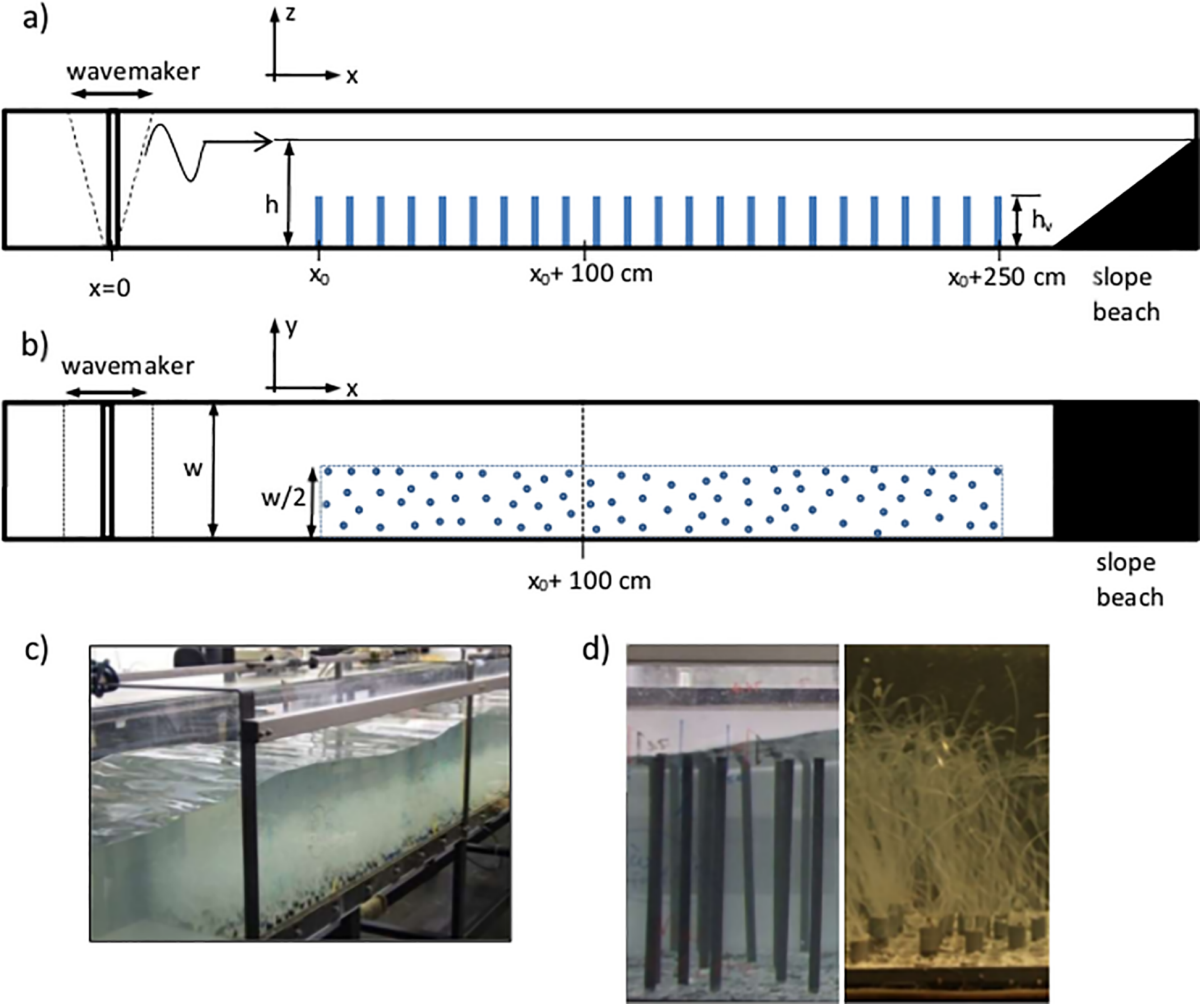
a) Scheme of the side view of the experimental setup. h represents the water height, h_v_ the vegetation height. b) Scheme of the top view of the experimental setup. c) Photograph of the flume with the distribution of the simulated vegetation. d) Photograph of the plants in the two vegetation models used (rigid and flexible).

The research was carried out in a laboratory flume (6 m long, 0.5 m wide and 0.5 m deep) with a mean water height, h, of 0.3 m (Fig 1A). The flume was equipped with a vertical paddle-type wavemaker at the entrance. The vertical paddle was driven by a variable-speed motor that operated at a frequency of 1.2 Hz. This frequency was chosen to align with previous work [8,9,23,46], and induced 1.03 m wavelengths, corresponding to transitional water waves which are typical in regions dominated by aquatic vegetation. A plywood beach (slope 1:3) was placed at the end of the flume and covered in foam to better attenuate incoming waves, thus ensuring that wave reflection was less than 10% of the incoming wave [8,13]. For further details of the experimental setup see Pujol et al [8]. We defined the longitudinal direction, x, to be zero at the position of the wavemaker in the longitudinal direction, the lateral direction, y, is zero at the centerline of the tank, and the vertical direction, z, is zero at the flume bed (Fig 1A and 1B).

To study edge effects, half the base of the flume was covered with a vegetation plant model (Fig 1B) and the other half was kept free of plants. For this purpose, the base of the flume was covered with 1-cm thick PVC boards perforated with 1cm in diameter holes, in which the plants were placed (Fig 1C). In this study we used two vegetation models, rigid and flexible (Fig 1D) with a height of h_v_ = 14 cm, and constructed following the details from Pujol et al [8]. The canopy model was placed 1 m away from the wavemaker. Rigid plants consisted of PVC cylinders 1 cm in diameter. Flexible plants consisted of polyethylene blades attached with a plastic band to a PVC dowel (2 cm long and 1 cm in diameter). Empty holes in the PVC boards were filled with dowels (1 cm long and 1 cm in diameter). The flexible model plants were constructed following the methodology of Pujol et al [47], so that they were dynamically and geometrically similar to *Posidonia oceanica* [8,38,48].

**Fig 2:**
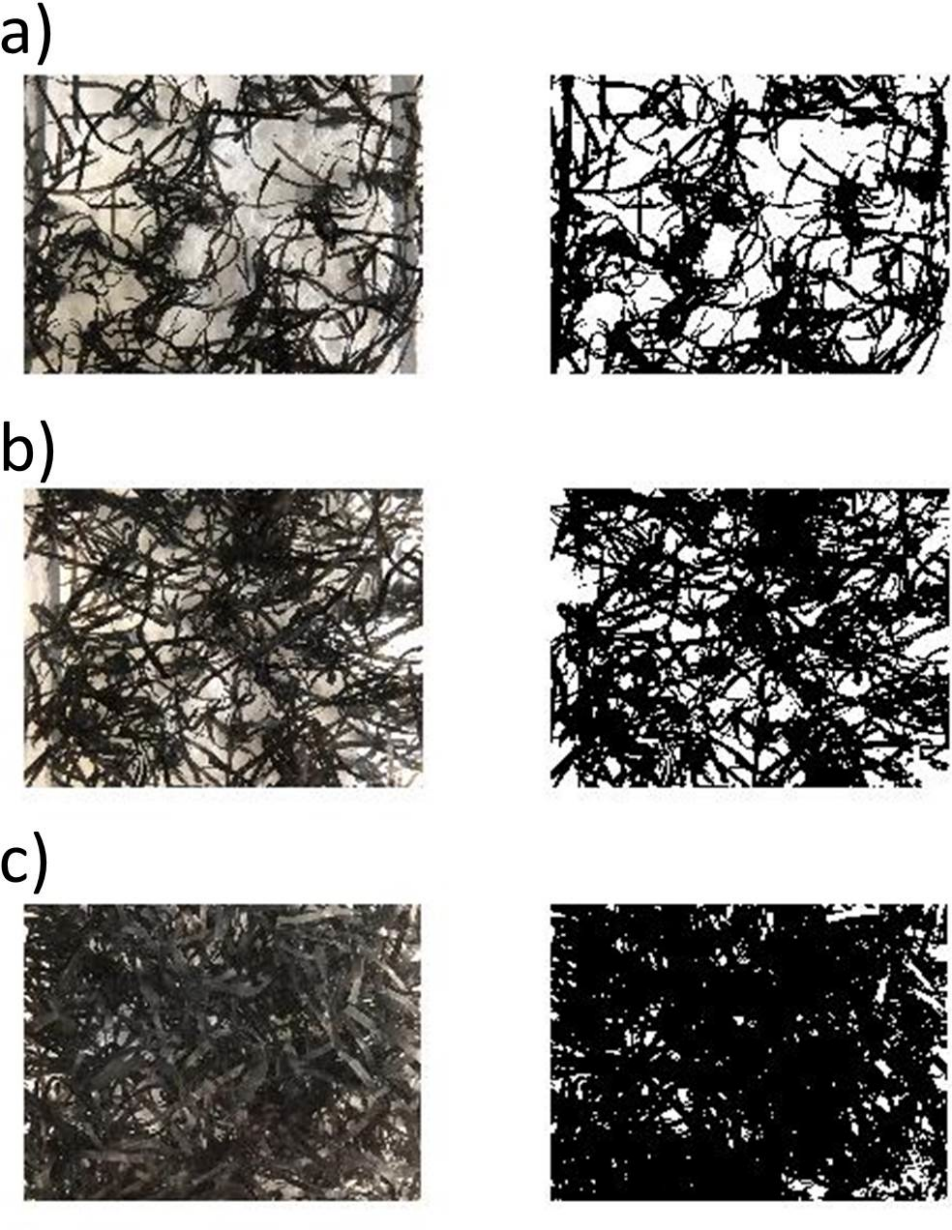
Top view photograph of the flexible canopy (left panels) and black and white digitized image using Matlab software (right panels) of canopy densities of a) SPF = 2.5%, b) SPF = 5% and c) SPF = 7.5%.

The density of the canopy was determined by the Solid Plant Fraction (SPF). The SPF can be defined [10] as the fraction of the bottom boundary occupied by stems SPF(%) = n_stems_A_stem_/A_total_×100, where n_stems_ is the number of stems, A_stem_ is the horizontal area of each stem (A_stem_ = πd^2^/4), where d is the plant diameter and A_total_ is the total horizontal area occupied by the canopy. Four SPF were used for the rigid canopy model (2.5, 5, 7.5 and 10%) and three for the flexible canopy model (2.5, 5 and 10%), with a canopy number density ranging from 320 to 1280 shoots/m^2^. The case for SPF = 0% was also studied. Furthermore, the case of a fully vegetated channel was also studied for all the SPF and for the two canopy models, rigid and flexible. The vegetation pattern for each SPF was created at random with a computer function. The cover of the flexible canopy was obtained from a binarized black and white photograph taken from the top of the canopy (Fig 2) and by using image analysis software with Matlab [10,49,50]. Flexible leaves were painted black and to increase the contrast the PVC bottom was substituted by a white board [50]. Photographs of each flexible canopy density were obtained and analyzed afterwards. The software distinguished between the black and white zones in order to calculate the area of the canopy. The cover obtained for a complete cover of the flume of flexible plants with SPF = 2.5%, 5% and 10%, was 40% (Fig 2A), 60% (Fig 2B) and 80% (Fig 2C), respectively. However, since the flume was half covered (Fig 1A), the cover considered was also half the value obtained for the full cover, i.e., 20%, 30% and 40%, respectively. For the rigid canopy, the cover coincided with the SPF for a full coverage of the flume. Since the flume was half covered, the cover for rigid canopies was half the SPF value in each case, i.e., 1.25%, 2.5%, 3.75% and 5% for SPF = 2.5%, 5%, 7.5% and 10%, respectively.

All measurements were taken in the central y–z plane, at x = x_0_+100 cm, where x_0_ is the position of the beginning of the stem distribution from the wavemaker for all SPF. The same position was also considered for SPF = 0%. Nineteen vertical velocity profiles were measured at different y-positions across the flume, thirteen within the vegetation (-23 cm, -21 cm, -19 cm, -17 cm, -15 cm, -13 cm, -11 cm, -10 cm, -9 cm, -7 cm, -5 cm, -3 cm and -1 cm) and six outside the vegetation (0, 3 cm, 5 cm and 7 cm, 9 cm and 11 cm), where y = 0 cm corresponded to the position of the edge of the vegetation across the width of the flume. For SPF = 0%, the velocity profile was made at the center of the flume (y = 0 cm). The measurements were taken with an Acoustic Doppler Velocimeter (16 MHz-ADV, SonTek Inc.). This instrument records (at 50 Hz) the three instantaneous velocity components at a single-point situated 5 cm from the probe tip using a sampling volume of 0.09 cm^3^. The ADV was placed in the flume in a downward looking configuration and connected to a PC with data acquisition software. The ADV instrument was configured to sample over 10-minute intervals (30,000 recordings per sampling interval).

The ADV was mounted in a frame and velocity profiles measured from z = 1 to 20 cm from the bottom of the flume, with a vertical resolution of 2 cm. Velocity measurements near the surface were limited by both wave shape and the 5-cm distance between the ADV sensor and the ADV sampling volume. To avoid spikes, beam correlations lower than 80% were removed. At two vertical positions (z = 8 cm and z = 20 cm above the bottom) low correlation was obtained. These ‘weak spots’ occurred when the first pulse emitted from the ADV was reflected at the bottom of the flume and met the second pulse within the sampling volume in time and space [8]. As the time lag between pulses depends on the velocity range, the ADV operational range was changed for these points (SonTek YSI, Acoustic Doppler Velocimeter Technical Instrumentation).

To obtain valid data acquisition within the canopy, a few stems were removed to ensure the ADV beam was not blocked and the acoustic receivers and transmitter performed properly [51,52]. To test the effect the ‘hole’ had on the ambient hydrodynamics, velocities were measured half a centimeter above the top of the canopy, both within and outside the hole. A 3% difference in velocities between ‘hole’ and ‘no hole’ canopies was observed at the highest SPF, while only 1% difference was observed at the lowest SPF. We therefore concluded that the ‘hole’ made minimal modifications to the ambient hydrodynamics.

## Method of analysis

In oscillatory flows the instantaneous velocity u can be decomposed into the time-averaged velocity (U_c_), orbital velocity (U_w_) and turbulent velocity (u’) components as:

$$u=U_{c}+U_{w}+u^{\prime }.$$ (1)

The above decomposition was made by using a phase-averaging technique [8,13] and the Hilbert transform was used to average oscillatory flow velocities with a common phase (φ). The velocity readings were binned into different phases as described by Pujol et al [9]. The root mean square of u(φ) was then defined as the orbital velocity U_w,rms_ (hereafter denoted U_w_) as:

$$U_{w,rms}=\sqrt{\frac{1}{2\pi }\int_{0}^{2\pi }{\left(u(\varphi )-U_{c}\right)}^{2}d\varphi }.$$ (2)

To calculate the turbulent kinetic energy (TKE) profile for stationary velocity records, the instantaneous velocities (u, v, w) at each sampling point were decomposed into the sum of time-averaged velocities (U_c_, V_c_, W_c_), orbital velocities (U_w_, V_w_, W_w_) and the turbulent components (u’, v’, w’) as described in Eq (1). The TKE was calculated as:

$$TKE=1/2(\bar{{u\prime }^{2}}+\bar{{v\prime }^{2}}+\bar{{w\prime }^{2}}).$$ (3)

where $\bar{{u\prime }^{2}},\bar{{v\prime }^{2}}\;and\;\bar{{w\prime }^{2}}$ are the time-average of the squared instantaneous turbulent velocities on the three axes, x, y and z, respectively.

## Results

**Fig 3:**
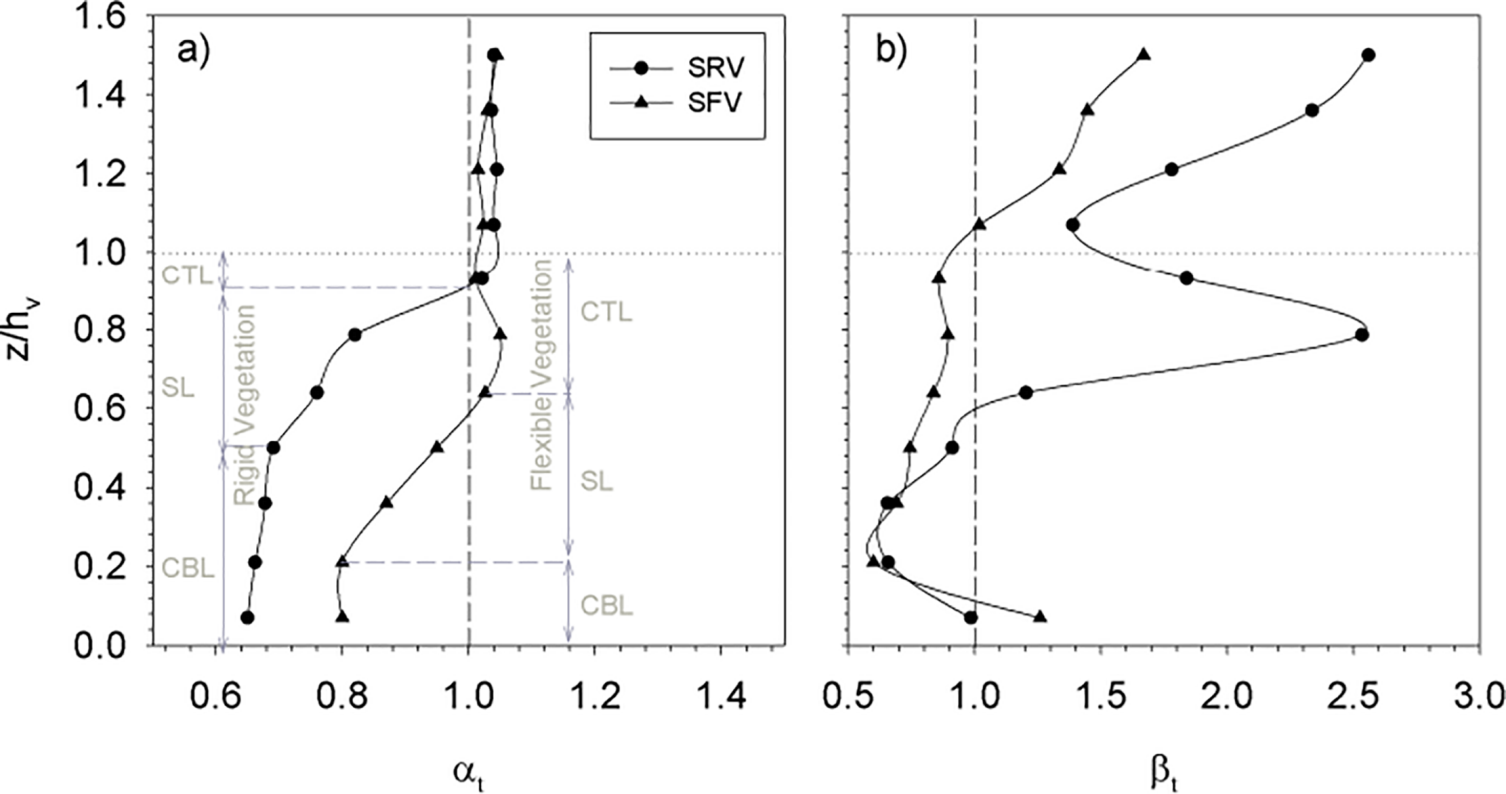
a) α_t_ profiles at y = 0 cm for the full vegetation case carried out at SPF = 10% for both rigid (SRV) and flexible (SFV) plants. b) β_t_ profiles at y = 0 cm for the full vegetation case carried out at SPF = 10% for both rigid and flexible plants. Vertical dashed lines represent the ratio α_t_ = β_t_ = 1. Horizontal dotted lines represent plant height. The vertical axis is the non-dimensional height z/h_v_ above the flume bed. Grey dashed lines represent the vertical layers occupied by the canopy. CTL = canopy top layer, SL = shear layer, CBL = canopy bottom layer.

The ratio between the wave velocity, U_w_ for the fully vegetated case and the case without plants (U_w,wp_), was calculated for both rigid and flexible model canopies for the SPF = 10%. The profiles of α_t_ = U_w_/U_w,wp_ for both rigid and flexible canopy models are presented in Fig 3A. From the vertical profiles, two distinct zones can be seen: the zone above the canopy and the zone within the canopy. For both rigid and flexible plants, in the above-canopy zone, the wave velocity ratio was slightly above 1. Within the canopy three layers could be differentiated: a) a *canopy top* layer, b) a *shear layer* situated below the canopy top layer and c) a *canopy bottom layer* below the shear layer. The extent of these layers depended on whether plants were flexible or rigid. In the canopy top layer, α_t_ remained similar to that measured in the zone above the canopy. This layer was thinner (about 1 cm thick) for the rigid canopy model than for the flexible canopy model (about 5 cm thick). Within the shear layer, α_t_ decreased gradually with depth, with Δα_t_/Δz = 0.06 cm^-1^ for the rigid vegetation and Δα_t_/Δz = 0.04 cm^-1^ for the flexible vegetation. This layer was 7 cm thick for both the rigid canopy model and the flexible canopy model and situated below the canopy top layer. In the canopy bottom layer, α_t_ was nearly constant with depth down to the bottom. In this layer, α_t_ attained its minimum value. This layer was 6 cm thick for the rigid canopy model and 3 cm thick for the flexible canopy model. Within the canopy, the wave velocity attenuation was greater for the rigid canopy model than for the flexible canopy model (Fig 3A). The ratio between turbulent kinetic energy (TKE) under full canopy coverage (TKE) and without plants (TKE_wp_) is presented in Fig 3B as β_t_ = TKE/TKE_wp_. For the flexible canopy model, β_t_ decreased with depth except for the measurement closest to the bottom (at z = 1 cm, Fig 3B). This increase can be attributed to the experimental setup of the flexible plant model that had a small dowel at the base to fix the flexible leaves of the plants. In the above canopy zone, the β_t_ was greater for the rigid canopy than for the flexible canopy model. At the rigid canopy top layer, β_t_ presented a maximum, and decreased again in the shear layer. Close to the bottom (at z = 5 cm), both rigid and flexible canopy models had similar β_t_.

**Fig 4:**
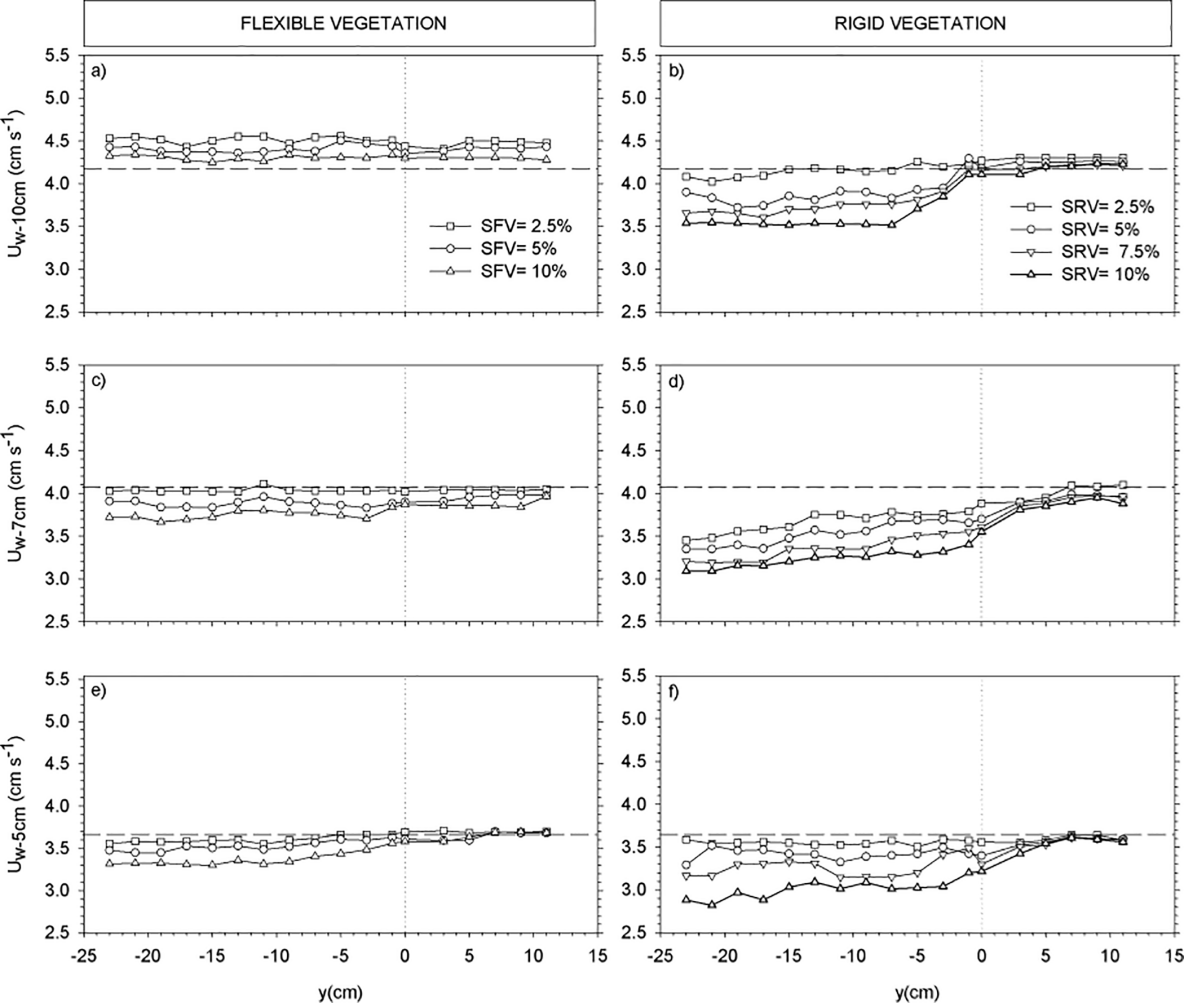
Wave velocity transects across the width of the flume (y-axis) for: z = 10 cm for flexible (a) and rigid (b) canopy models, for z = 7 cm for flexible (c) and rigid (d) canopy models, and z = 5 cm for flexible (e) and rigid (f) canopy models. Horizontal dashed lines correspond to wave velocities for the experiment without plants. Vertical dotted lines correspond to the y-position of the edge of the vegetation. Negative y values correspond to the region covered with plants.

**Fig 5:**
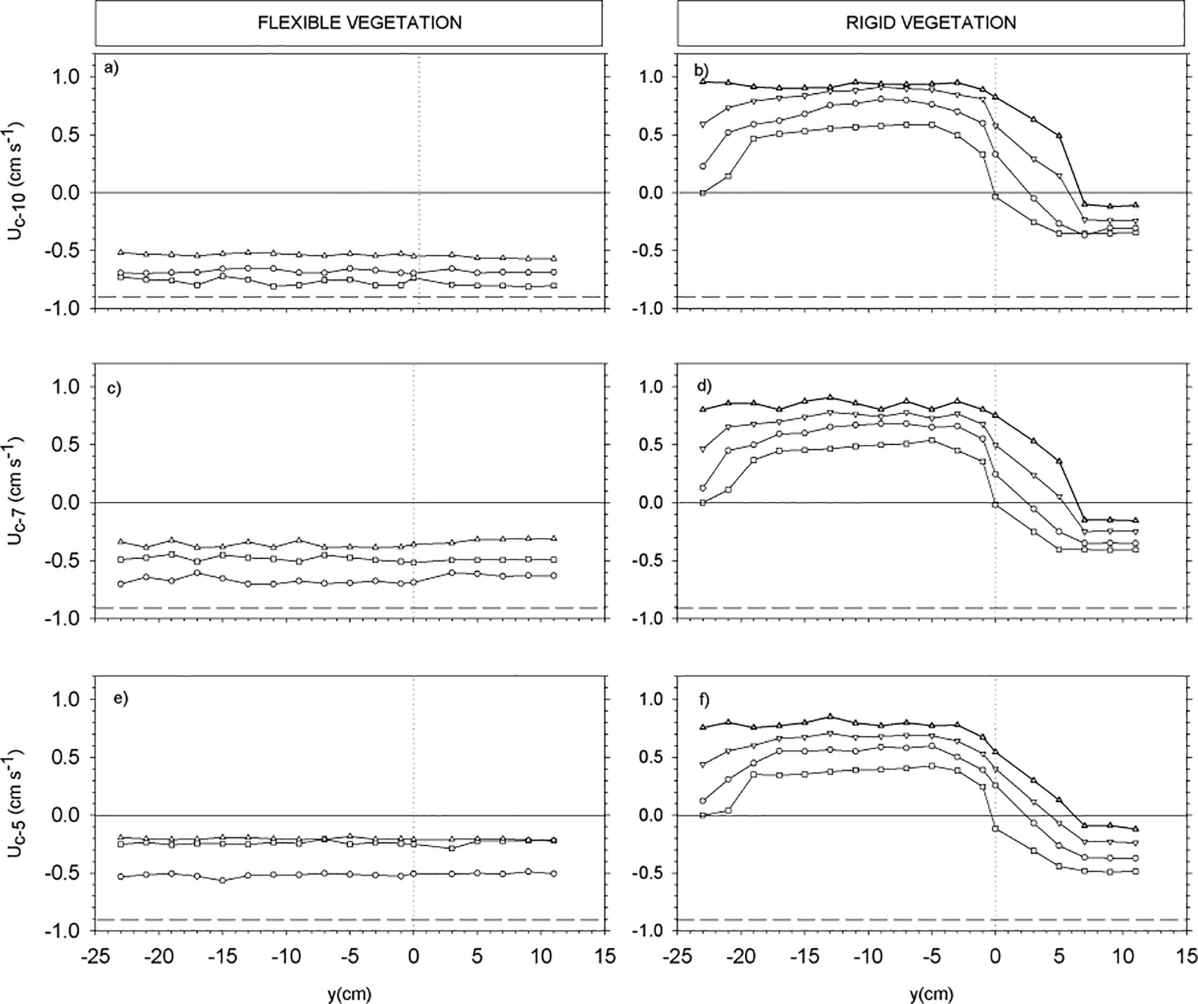
Mean velocity transects across the width of the flume (y-axis) for: z = 10 cm for flexible (a) and rigid (b) canopy models, for z = 7 cm for flexible (c) and rigid (d) canopy models, and z = 5 cm for flexible (e) and rigid (f) canopy models. Positive values indicate flow towards the beach slope while negative values indicate that the flow is directed towards the wavemaker. Horizontal dashed lines correspond to mean velocities for the experiment without plants. Vertical dotted lines correspond to the y-position of the edge of the vegetation. Negative y values correspond to the region covered with plants.

**Fig 6:**
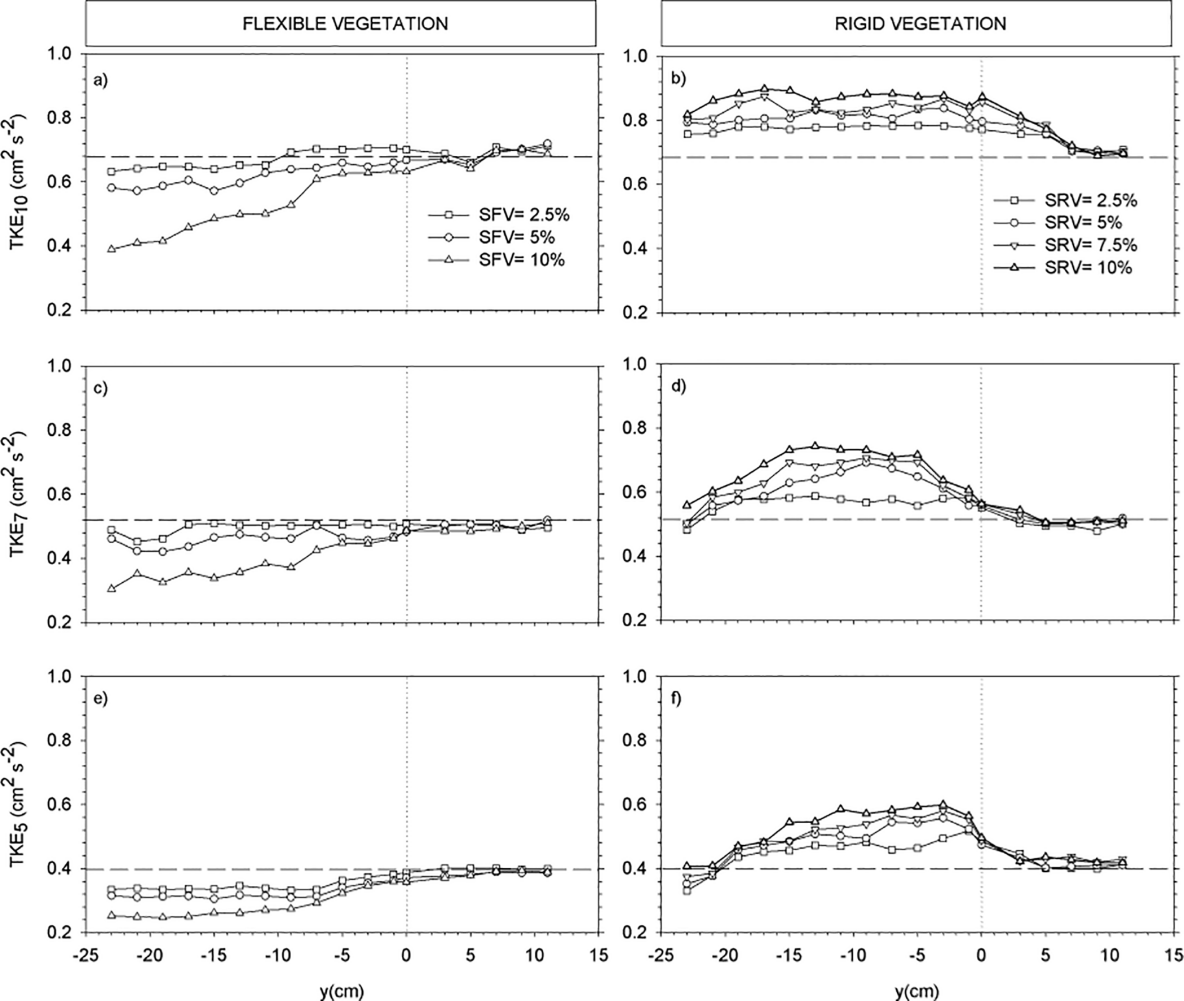
Turbulent kinetic energy transects across the width of the flume (y-axis) for: z = 10 cm for flexible (a) and rigid (b) canopy models, for z = 7 cm for flexible (c) and rigid (d) canopy models, and z = 5 cm for flexible (e) and rigid (f) canopy models. Horizontal dashed lines correspond to turbulent kinetic energies for the experiment without plants. Vertical dotted lines correspond to the y-position of the edge of the vegetation. Negative y values correspond to the region covered with plants.

For the cases with a partial vegetated canopy, we investigated the transversal evolution of U_w_, U_c_ and the TKE in the three canopy layers. To this purpose, three depths were considered within each layer. The depths z = 5 cm, 7 cm and 10 cm were considered to describe the distribution of the wave velocity, the mean flow and the turbulent kinetic energy across the edge of the vegetation. Transects of U_w_ (Fig 4), U_c_ (Fig 5) and TKE (Fig 6) across the y direction were plotted for each depth for both rigid and flexible plants at the selected y-locations (see the methods section). The position of the edge of the canopy was situated at y = 0 cm, therefore negative y-values indicate the region within the vegetation model and positive y-values indicate the region without plants. At z = 10 cm, U_w_ was slightly above U_w,wp_ for the flexible canopy model (Fig 4A) showing the behavior expected in the canopy top layer. In contrast, for the rigid canopy model, U_w_ at z = 10 cm was already below the U_w,wp_ (Fig 4B), as expected given that z = 10 cm corresponded to the sheared layer for the rigid canopy model. The degree of wave velocity attenuation increased with SPF. For the rigid canopy, U_w_ and was close to U_w,wp_ near the edge within the canopy, and afterwards decreased further towards the inner part of the canopy. Within the canopy at z = 10 cm, the TKE reached a minimum at y = -7 cm and remained constant within the canopy. The depth z = 7 cm was within the sheared layer for both rigid and flexible canopy models. The rigid canopy attenuated waves more than the flexible canopy did, as evidenced by the higher U_w_ in the flexible canopy model (Fig 4C) compared to the rigid canopy model (Fig 4D). At z = 5 cm, U_w_ for the flexible canopy model was less than U_w,wp_ (Fig 4E); this was also observed for the rigid canopy model (Fig 4F). The impact SPF had on wave velocity attenuation was greater for the rigid canopy model than for the flexible canopy model. Outside the region covered with flexible vegetation, U_w_ was close to U_w,wp_ (Fig 4A, 4C and 4E). For rigid plants, U_w_ outside the vegetated region increased gradually with distance from the canopy edge, with a tendency towards the value of U_w,wp_ (Fig 4D and 4F).

At z = 10 cm and within the region covered by plants, U_c_ for the flexible vegetation was smaller than U_c,wp_, but still negative, and flowed in the same direction, i.e., opposite to the wave direction (Fig 5A). The denser the vegetation, the smaller the U_c,_, however, U_c_ was the same independently of whether the y-position was outside or inside the region covered by flexible plants. In contrast, U_c_ within the rigid vegetation shifted to positive values, indicating that the flow in this region was in the opposite direction to that measured in the experiment without plants (Fig 5B). U_c_ outside the rigid vegetated region gradually decreased with distance from the edge, tending towards the U_c_ measured in the experiment without plants.

The transects of TKE are presented in Fig 6. At z = 10 cm, TKE within the flexible vegetation decreased compared to TKE measured outside the vegetation. The greatest decrease in TKE was measured at the highest SPF (i.e.10%) (see Fig 6A). For the sparser canopy of 2.5% SPF, the TKE at z = 10 cm was close to TKE_wp_. At the edge, the TKE was close to TKE_wp_ for all the SPF studied. No reduction in TKE was found outside the vegetated region. In contrast, the TKE within the rigid canopy increased compared to the TKE measured outside the vegetation. The greater the SPF, the higher the TKE inside the vegetation. The TKE at z = 10 cm was nearly constant across the rigid vegetated region. Outside the vegetation, TKE decreased and gradually approached TKE_wp_. The same patterns of TKE distribution were found at z = 7 cm (Fig 6C and 6D) and at z = 5 cm (Fig 6E and 6F). However, for the rigid vegetation and well inside the vegetated region (from z = -18 cm to z = -23 cm) and at both z = 7 cm and z = 5 cm, the TKE decreased gradually and in some cases at z = 5 cm was less than TKE_wp_ (Fig 6D and 6F).

## Discussion

The wave velocity, the mean flow velocity and the turbulent kinetic energy at the longitudinal edge of a simulated meadow have been found to be modified by the flexibility and density of the lateral vegetation for a constant submergence ratio of h_v_/h = 0.47. The longitudinal edge is thus found to be a region of transition where local hydrodynamics depend on the properties of the canopy. Our results demonstrate consistent modification of the mean flow, the turbulent kinetic energy and the wave velocity across the longitudinal edge of a canopy, suggesting that seagrass canopies have the potential to act as ecosystem engineers and modify local edge hydrodynamics at edges.

## Longitudinal edge boundary layers inside canopies: Rigid versus flexible plants

Rigid canopies were found to modify hydrodynamics at longitudinal edges in a different manner to flexible canopies. Wave velocities, U_w_, penetrated more deeply into flexible canopies than rigid canopies and, for both canopy types, wave velocities became constant inside the canopy. The attenuation of the wave velocity with distance inside the flexible canopy, induced a reduction in the mean flow velocity, U_c_ (Fig 5A, 5C and 5E). However, within the rigid canopy, U_c_ shifted its direction producing a shear layer at the longitudinal edge of the canopy (Fig 5B, 5D and 5F) that resulted in an increase in the TKE. As measured by Pujol et al [8] under full canopy cover (i.e., without gaps), our experiments demonstrated that mean velocities in the top 5 cm of the water column, well above the top of the canopy, flowed towards the beach for both rigid and flexible canopies. Below this layer, but still above the canopy, a return flow directed away from the beach was measured. The direction of the mean velocities inside the canopy was different for flexible and rigid vegetation. Within the rigid canopy, the flow reversed again (positive), which also agreed with Pujol et al [8]. The mean current velocities were also positive over the bare sediment immediately adjacent to the rigid canopy. This pattern of flow within the canopy was different to that found within the flexible canopies. Inside the latter, the mean velocity aligned with flows above the canopy, albeit except for a thin layer at the canopy base. Again, this mirrored the measurements described by Pujol et al [8,9] in an experiment done with fully canopy cover.

In summary, a key feature measured by our experiments was that for the flexible canopy, the direction of the mean flow inside the canopy was the same as outside the canopy (both above and to the side). In contrast, there was a reversal of flows for the rigid canopy (inside versus outside). This produced a horizontal shear layer at the longitudinal edge of the canopy; something which had previously been observed by Nezu and Onitzuka [45] and White and Nepf [53] using a partially vegetated flume with a rigid canopy, but in their case it was under a unidirectional flow.

This shear layer at the rigid longitudinal canopy edge triggered an increase in TKE with distance into the canopy, this effect was accentuated with increasing SPF. This contrasted with the trend of decreasing TKE across the flexible longitudinal canopy edge (see Fig 6A, 6C and 6E), and again this effect was accentuated with increasing SPF.

### Hydrodynamic regions across the longitudinal edge of a submerged canopy

**Fig 7:**
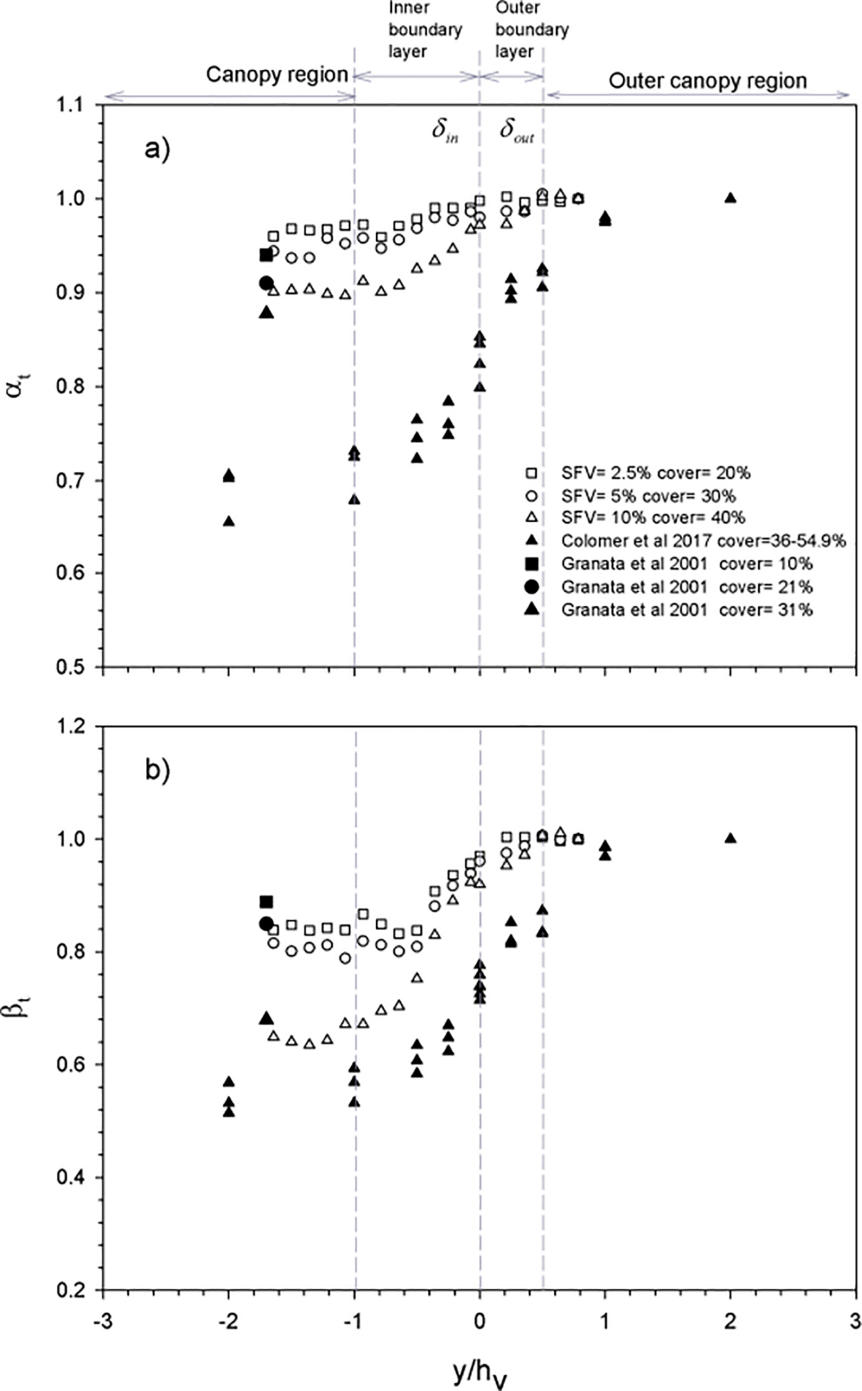
a) α_t_ transects across the width of the flume (y-axis) scaled as y/h_v_ at z = 5 cm above the bottom and for flexible vegetation. b) β_t_ transects across the width of the flume (y-axis) scaled as y/h_v_ at z = 5 cm above the bottom and for flexible vegetation. Data from Granata et al [11] and Colomer et al [21] are included. Vertical dashed lines show the limits of the four different zones across the width of the flume, the canopy region, the inner boundary region, the outer boundary layer and the outer canopy region.

The attenuation of both the wave velocity (α_t_, Fig 7A) and the turbulent kinetic energy (β_t_, Fig 7B) versus the non-dimensional distance y/h_v_ have been plotted together with the data from Granata et al [11] and Colomer et al [21], both obtained at edges of coastal canopies. During their field survey, Granata et al [11] studied a seagrass canopy with a longitudinal edge, i.e., with the main flow direction aligned with the edge. Colomer et al [21] did not specify the main flow direction in any of their surveys, however, the main axis of their gaps were perpendicular to the shore and the predominant wave field in this region is also perpendicular to the shoreline. Therefore, in Colomer et al [21], longitudinal edges could be considered a good approximation. Granata et al. [11], made the velocity measurements within the canopy at z/h_v_ = 0.25. In this present study, measurements were taken at z/h_v_ = 0.36. Therefore, both studies took their measurements within the sheltered layer (Fig 3A), where the wave velocity attenuation is nearly constant. Colomer et al. [21] made the measurements at z/h_v_ = 0.42, slightly above the sheltered layer but still at a depth where the wave velocity remains attenuated (Fig 3A). Combining these field data with our laboratory data, α_t_ and β_t_ show that four regions can be distinguished across the longitudinal edge of the canopy. A region outside the canopy (the outer canopy region) where both α_t_ and β_t_ are close to 1, indicating a negligible velocity attenuation of both α_t_ and β_t_. An outer boundary layer where both α_t_ and β_t_ are below 1 and decreasing gradually towards the longitudinal edge of the canopy, and both α_t_ and β_t_ decreasing with the canopy cover. Results from Granata et al [11] and Colomer et al [21] are in accordance with the results presented here. The inner boundary region, where α_t_ and β_t_ decrease with distance from the longitudinal edge of the vegetation up to a limit, already in the canopy region with a constant value of α_t_ and β_t_ at a distance y that was around the plant height (δ_in_ = h_v_); this distance indicates the width of the inner boundary layer inside the canopy. For the same canopy densities, Granata et al [11] found similar α_t_ and β_t_ values as those found in the canopy layer at a non-dimensional distance of -1.7 y/h_v_ within the canopy (Fig 7).

The change of α_t_ and β_t_ across the longitudinal edge of a canopy showed that there is a gradual transition in the hydrodynamics from outside the canopy to the inner canopy region. Flexible canopies offer a region where both the turbulent kinetic energy and the wave velocity decrease from the edge of the canopy towards the inner part of the canopy. The boundary is a region in between the inner canopy and the bare soil. The greater levels of TKE and wave velocities compared to those in the inner canopy might produce a greater sediment resuspension and therefore an increase in the turbidity at the boundary, reducing light availability. A reduction in light availability may contribute to producing lower canopy densities at canopy edges when compared to the inner canopies. This effect could help explaining why the biomass of a canopy increases smoothly, rather than abruptly, with distance from the longitudinal canopy edge [54]. Another important factor to consider in determining the biomass of a seagrass across the edge of a canopy is its age. The regions situated at the edge are the younger parts of a seagrass, with a low above- and belowground biomass, while the innermost part of the canopy is the oldeest part with high aboveground and belowground biomass [55].

*P*. *oceanica* canopies with canopy covers in the same range as those used here [11] presented similar α_t_ and β_t_ values to those obtained in the present study and have been plotted in Fig 7A and 7B, respectively. In our experiments, the flexible canopy of SPF = 2.5% (cover = 20%) produced a lower wave velocity attenuation than that for SPF = 10% (cover = 40%). α_t_ and β_t_ values for denser *P*. *oceanica* canopies with greater covers of 45% [21] have been also plotted in Fig 7A and 7B and presented greater wave velocity attenuations despite having equal shoots per m^2^. Therefore, results are in accordance with Paul and Amos [56], who stated that the extent of wave attenuation by the canopy is a function of the canopy density. Luhar et al [13] studied the wave velocity attenuation by a flexible vegetation in the laboratory with a complete cover of the flume. They found that, at z = 5cm above the bottom, the wave velocity attenuation was α_t_ = 0.92 for a canopy density of 1200 shoots m^-2^. This result is in accordance with the results found in the present work, where in the inner part of the canopy, the wave velocity attenuation reaches α_t_ = 0.9 for a canopy density of 1280 shoots m^-2^. In a *Posidonia oceanica* meadow, Infantes et al [57] found a wave velocity attenuation ranging from α_t_ = 0.8 when comparing the wave velocity in the inner canopy with the wave velocity in the location situated closest to a nearby edge of the canopy. For the furthest station from the longitudinal edge, the wave velocity attenuation reached a minimum of α_t_ = 0.6 when compared to the station near the edge. Paul and Amos [56] also stated that the highest wave frequencies were more attenuated than the lowest ones. We did not observe such behavior when comparing laboratory (with f = 1.2Hz) with field (with f~0.25Hz) measurements. However, since different α_t_ and β_t_ values were obtained for different canopy covers, but with similar canopy number densities, the canopy cover may be the best indicator of the differences observed in the hydrodynamics of canopy edges.

**Fig 8:**
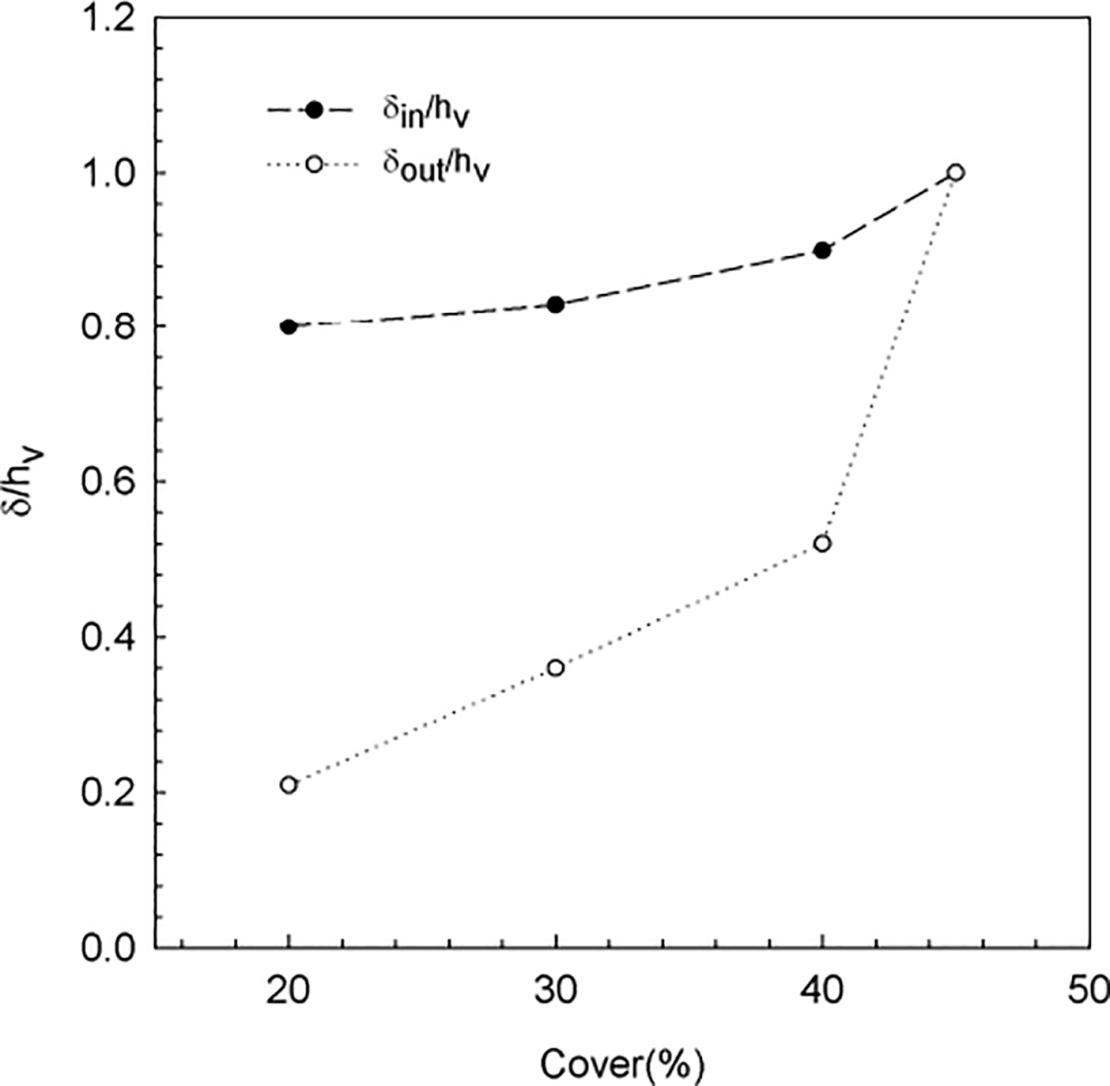
Non-dimensional length (δ/h_v_) of the inner and outer boundary layers versus the canopy cover.

The length scale of the inner boundary (δ_in_) was calculated as the length from the longitudinal edge at which U_w_ and TKE attained a constant value that remained constant within the canopy thereafter (Fig 7). Therefore, δ_in_ was the y value at which the linear trend of both TKE and U_w_ in the inner boundary layer intercepted the horizontal constant value of U_w_ and the TKE in the canopy region. The length scale of the outer boundary (δ_out_) was calculated as the length from the longitudinal edge at which U_w_ and TKE attained a constant value equal to U_w,wp_ and TKE_wp_, respectively. Thus, δ_out_ was the y value at which the linear trends at the outer boundary layer of both U_w_ and TKE intercepted the constant value of U_w,wp_ and TKE_wp_, respectively. This calculation was done at the measurement point, i.e., 1 m from the beginning of the vegetation. The non-dimensional scales δ/h_v_ for both the inner and the outer boundary layers versus the cover for the results of the present study and those from Colomer et al [21], for a mean canopy cover of 40% (Fig 8) have been plotted. δ/h_v_ for the inner boundary layer presented a slight increase with the canopy cover from 0.9 for the experiments carried out in this study to δ/h_v_ = 1 for the field case carried out by Colomer et al [21]. The inner canopy length scale represents the length up to which the canopy is affected by the nearby bare soil, i.e., where high levels of turbulence and wave velocities prevail. Characteristic values of the scale of the inner boundary layer have been found by other authors studying the biomass of a *Zostera muelleri* seagrass. They found that most of the change in the biomass occurred in the first 0.5 m from the edge of the canopy. Since *Zostera muelleri* seagrass has a mean leaf length of 0.5 m [58] and, based on the biomass change, δ/h_v_ was close to 1. δ/h_v_ for the outer boundary layer increased markedly with the canopy cover.

In comparison with the flexible canopy, a longer boundary layer length scale was obtained for the rigid canopy, coinciding also with an increase in the turbulent kinetic energy and a shift in the mean flow from positive values within the canopy towards the bed where the mean flow reaches negative values. The shift in the mean current direction might be similar to the streaming flow produced at boundaries in unidirectional flows [45], which has been also related to an increase in the turbulent kinetic energy at the boundary. However, in a unidirectional flow regime, the flow velocities are usually one order of magnitude greater [53] than the mean flow velocities associated with the wave field that have been found in the present study. The increase in the turbulent kinetic energy might produce erosion of the bed at the longitudinal edges, like those found in the field patches under unidirectional flow for leading canopy edges and longitudinal edges [14]. Bouma et al [14] also found that high canopy densities produced greater erosion at the leading edges than low canopy densities did. In the present study, this might be explained by the larger boundary layer length scale provided by denser canopies compared to low canopy densities. High levels of turbulent kinetic energy are also associated to a greater sediment resuspension [4,59,60].

**Fig 9:**
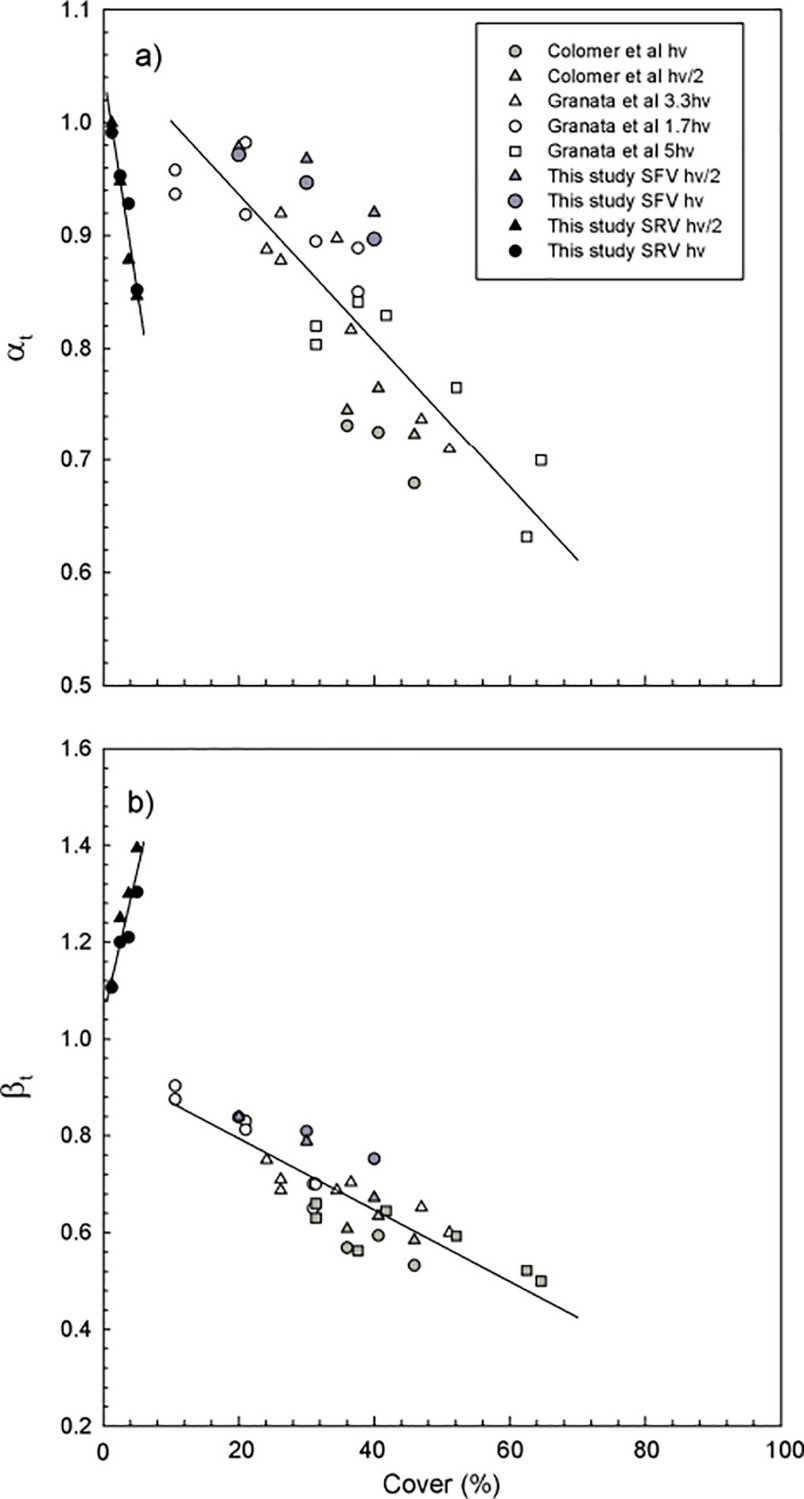
a) Relationship between α_t_ and the canopy cover for flexible vegetation (with a linear fit α_t_ = -0.651×10^-2^cover+1.066, r^2^ = 0.684, 99% confidence) and for rigid vegetation (with a linear fit α_t_ = -3.889×10^-2^cover+1.046, r^2^ = 0.946, 99% confidence). b) Relationship between β_t_ and the canopy cover for flexible vegetation (with a linear fit β_t_ = -0.739×10^-2^cover +0.943, r^2^ = 0.689, 99% confidence) and for rigid vegetation (with a linear fit β_t_ = 5.990×10^-2^cover +1.047, r^2^ = 0.825, 99% confidence).

The results obtained for α_t_ and β_t_ in the longitudinal edge of laboratory simulated canopies were plotted versus the canopy cover in Fig 9A and 9B, respectively. Results for α_t_ and β_t_ obtained in field canopies by Granata et al [11] and Colomer et al [21] were also included for completion in the analysis. The canopy characteristics for all the studies considered are listed in Table 1. Data considered corresponded to y-positions inside the canopy greater than h_v_/2 from the longitudinal edge of the canopy, i.e., falling in the canopy region or close to the canopy region. Results for flexible canopies obtained in the present study, together with those of Colomer et al [21] and Granata et al [11], showed a linear decrease of α_t_ with the canopy cover, with a slope of -0.651×10^−2^ (Fig 9A). However, the rigid canopies in the present study presented a stronger linear decrease of α_t_ with the canopy cover, with a slope of -3.889×10^−2^ (Fig 9A), in accordance with the fact that rigid plants produced higher wave velocity attenuations (Fig 4).

**Table 1:** Solid plant fraction range (in %), canopy type, shoots per square meter, cover (in%) for the different experiments carried out (in %) in this study and also for those used for the analysis and comparison.

SPF(%)	Canopy type	Shoots/m^2^	Cover (%)	Font
2.5–10	simulated, flexible vegetation	320–1280	20–40	This study
-	*P*. *oceanica*	231–311	36–54.9	Colomer et al (2017)
-	*P*. *oceanica*	50–200	10–31	Granata et al (2001)

El Allaoui et al [36] studied the effect wave penetration has in lateral rigid vegetation near to a longitudinal gap using a similar setup like that used in this present study. They found high wave velocity attenuations of α_t_ = 0.62 and α_t_ = 0.67 for canopy densities of 10% with gap widths of 12.5 cm and 18.75 cm. The greater wave velocity attenuations in their case compared to those obtained here, might be attributed to the small gap used in their studies compared to the size of the bare bed (of 25 cm) used in the present experiment. The lateral vegetation situated at the two sides of the gap, protected the small gap used by El Allaoui et al [36]. And, conversely, the gap in their case was probably too small to allow the same wave penetration within the lateral vegetation as that in the present study. Folkard [38] also found that the flow penetration within both the gap and the nearby lateral vegetation depended on gap size. The vertical wave attenuation parameter (α_w_ = U_w,5cm_/U_w,∞_, where U_w,∞_ is the wave velocity far above the canopy and U_w,5cm_ is the wave velocity within the canopy at 5 cm above the bottom) was calculated for the cases studied in the laboratory (Table 2). The values obtained for α_w_ are in accordance with those obtained by Pujol et al [8] for the same canopy densities and the same range of S/d and A_∞_/S (where S = 1/N^1/2^-d is the plant-to-plant distance, A_∞_ = U_∞_/(ωS) is the orbital wave excursion length scale and ω = 2πf is the wave frequency in rad s^-1^) and are also in accordance with the model proposed by Lowe et al [12].

**Table 2:** Solid plant fraction (SPF), vertical wave velocity attenuation (α_w_), ratio between the orbital excursion length (A_∞_) and the plant-to-plant distance (d), ratio between the plant-to-plant distance (S) and the stem diameter (d) and vegetation type (flexible or rigid).

SPF (%)	α_w_ = U_w,5cm_/U_w,∞_	A_∞_/S	S/d	Vegetation type
2.5	0.86	0.12	4.6	Flexible
5	0.82	0.19	3	Flexible
7.5	0.79	0.25	2.2	Flexible
2.5	0.83	0.12	4.6	Rigid
5	0.81	0.19	3	Rigid
7.5	0.76	0.25	2.2	Rigid
10	0.71	0.31	1.8	Rigid

Flexible canopies also produced a linear decrease of β_t_ with the canopy cover with a slope of -0.739×10^−2^ (Fig 9B). In contrast, rigid canopies showed a linear increase of β_t_ with the canopy cover with a slope of 5.990×10^−2^ (Fig 9B). Denser *P*. *oceanica* canopies have been documented with covers from 25% to 100% [61]. From the linear decrease of α_t_ and β_t_ obtained here, a flexible canopy cover of 100% would produce a maximum wave velocity attenuation of 58.5% (i.e. α_t_ = 0.415) and a maximum turbulent kinetic energy attenuation of 79.7% (β_t_ = 0.203). Those values would represent the highest attenuation levels achieved by flexible *P*. *oceanica* canopies. In addition, the canopy cover, rather than SPF, seems to better predict both the wave and the turbulent kinetic energy attenuations within a canopy.

The differences observed between rigid and flexible vegetation highlight that an experimental simulated rigid canopy does not reproduce the hydrodynamics experienced by a flexible canopy. Therefore, as noted by other authors [13,36,62], plant flexibility is a fundamental parameter to be carefully considered when attempting to mimic the environment around aquatic plants in the laboratory. In this present study, the impact on the hydrodynamics of flexible and rigid canopies was studied. Rigid and flexible plants present different hydrodynamics at the canopy edges which also has an impact on the bare soil nearby. In the field, the bending of seagrass plants will vary with season; when leaves are shorter in winter they will behave more like rigid stems whereas in summer, when the leaves are longer, they will bend easily, bringing their movement closer to that observed for flexible plants. Together with our results, this suggests that the hydrodynamic conditions inside the canopy, and along its edges, will vary seasonally, not just in response to seasonal hydrodynamics, but also due to interaction between the life cycle of the seagrass and the modified hydrodynamics.
